# Diagnosis in neurodevelopmental disorders: time, dynamics, and response to intervention

**DOI:** 10.3389/fnins.2026.1858730

**Published:** 2026-08-28

**Authors:** Bertrand Schoentgen

**Affiliations:** 1 Child Neurodevelopment Unit, Aloïs Center, Paris, France; 2 Université d’Angers, Angers, France

**Keywords:** brain maturation, developmental time, developmental trajectories, diagnostic reasoning, early identification, longitudinal assessment, neurodevelopmental disorders, response to intervention

## Abstract

Neurodevelopmental disorders are defined within a developmental framework, yet diagnostic reasoning in clinical practice still relies largely on observations made at a single time point. This creates a tension: intervention is expected early, while diagnostic validity depends on how difficulties evolve over time. We argue that this tension reflects a limited integration of time into diagnostic reasoning. In the absence of validated biomarkers, clinical manifestations are not static. They emerge from interactions between brain maturation, environmental demands, and compensatory processes. Their meaning depends on how they change over time and respond to intervention. This perspective calls for a better account of developmental variability and the heterogeneity of trajectories, particularly in childhood. During this period, expectations change rapidly, and differences in maturation may lead to divergent interpretations of similar behaviors. Executive functions are discussed as one illustrative developmental marker because of their prolonged maturation, transdiagnostic nature, and sensitivity to contextual demands. Diagnosis is therefore better understood not as a one-time decision, but as a process grounded in developmental trajectories. Longitudinal observation, including repeated assessments and changes in response to structured environmental adjustments, provides critical information to distinguish transient developmental difficulties from more persistent neurodevelopmental constraints. This framework has implications for both clinical practice and research. It supports early identification and intervention, while emphasizing the need for structured longitudinal monitoring and stronger integration of temporal dynamics into diagnostic models. In neurodevelopment, diagnosis is not the reading of a state, but the interpretation of a trajectory.

## 1 Introduction

Neurodevelopmental disorders (NDDs) represent a major public health concern due to their high prevalence, long-term impact on cognitive, educational, and social functioning, and their substantial societal costs (Doshi et al., 2012; Schoentgen et al., 2025; Thapar et al., 2017). Advances in brain development research, together with increasing recognition of behavioral, emotional, and learning difficulties, have contributed to growing expectations for early identification, intervention, and diagnostic clarification. Requests for evaluation frequently arise when development difficulties interfere with schooling, social functioning, or family life, in healthcare systems where access to support and interventions often remains closely linked to diagnostic decisions. Clinicians may therefore be expected to establish categorical diagnoses at developmental stages when stability, significance, and future course of observed difficulties remain uncertain.

This issue may be illustrated by epidemiological observations in attention deficit hyperactivity disorder (ADHD), where discrepancies between lifetime diagnosis rates and current prevalence estimates highlight the influence of developmental dynamics on diagnostic outcomes. In the United States, approximately 15.5% of adolescents aged 12–17 have reportedly received a diagnosis of ADHD at some point, whereas current prevalence estimates are substantially lower, typically around 9%–11% (Danielson et al., 2024; Visser et al., 2014). Although particularly well-documented in ADHD, these issues are unlikely to be specific to this condition. Recent literature has highlighted substantial variability in diagnostic practices across neurodevelopmental disorders, suggesting that diagnostic outcomes remain influenced by contextual, procedural, and interpretative factors (Bishop et al., 2017; Norbury et al., 2016; Hull et al., 2020).

In the absence of validated biomarkers, and with increasing recognition of phenotypic heterogeneity and less prototypical clinical presentations across neurodevelopmental disorders, this issue becomes particularly salient. Clinical manifestations emerge within a context of gradual and asynchronous brain maturation, changing environmental demands, developmental variability, and compensatory processes that may modify their expression over time (Best and Miller, 2010; Casey et al., 2005; Diamond, 2013). Advances in neuroscience have emphasized both the plasticity of the developing brain and the sensitivity of neural circuits to early environmental influences (Johnson, 2011; Kolb and Gibb, 2014). Yet, the extent to which these temporal dimensions contribute to diagnostic interpretation remains insufficiently explicated and inconsistently incorporated into clinical reasoning and practice. This raises a central question: how should developmental time be integrated into clinical decision-making?

The present Perspective examines how developmental time may inform diagnostic reasoning in light of current knowledge regarding brain maturation, developmental variability, and heterogeneous developmental trajectories (Marschik et al., 2017; Prechtl, 2001). Executive functions are discussed as one illustrative developmental marker because of their prolonged maturation, transdiagnostic nature, and sensitivity to contextual and compensatory processes (Best and Miller, 2010; Diamond, 2013; Nigg, 2017). More broadly, this Perspective explores whether concepts often considered separately in clinical practice and research may be integrated within a developmental framework, with implications for both diagnostic reasoning and the interpretation of research cohorts.

## 2 The diagnostic paradox in neurodevelopmental disorders

The growing emphasis on early identification and intervention in neurodevelopmental disorders reflects a legitimate objective: the earlier vulnerabilities are recognized, the earlier appropriate support can be implemented, with the potential to influence developmental trajectories. Early intervention programs have demonstrated benefits across language, social functioning, and academic domains (Dawson et al., 2010), making early identification a priority in clinical practice, public health, and educational systems. However, this evolution reveals a mismatch in clinical reasoning. Early identification, often initiated by parents, teachers, or primary care providers, tends to generate an expectation of rapid diagnostic categorization, following a model inherited from other medical fields, where identifying a disorder leads directly to classification.

In neurodevelopment, this logic is currently limited by the absence of validated biomarkers. Diagnosis relies on the interpretation of behavioral and functional manifestations whose meaning depends on their evolution over time and under adapted environmental conditions during a period of rapid cognitive and socio-emotional changes (Johnson, 2011). This variability complicates the interpretation of early clinical signs: behaviors that appear atypical at one point may reflect transient variation, while others emerge only as environmental demands increase. Clinical expression thus reflects interactions between neurobiological vulnerabilities, partly genetically driven, compensatory capacities, and environmental conditions (Best and Miller, 2010; Diamond, 2013).

This leads to a diagnostic paradox: clinicians are encouraged to establish an early diagnosis to initiate intervention, while diagnostic validity depends on observing developmental trajectories over time (Johnson, 2011; Lord et al., 2020). This perspective aligns with recent recommendations emphasizing that support should be needs-led and should not be delayed by the absence of a formal diagnosis (Bishop et al., 2017; Lord et al., 2020; McGregor, 2020; NHS England, 2025). In practice, this tension may lead either to delaying intervention while awaiting greater diagnostic certainty, or to establishing an early diagnosis despite persistent uncertainty.

Although diagnostic criteria for most neurodevelopmental disorders are well-defined within current classifications, their implementation remains heterogeneous. Variability in age at diagnosis, access to services, thresholds for clinically significant impairment, and reliance on longitudinal observations has been described across developmental language disorder, developmental coordination disorder, autism spectrum disorder, and ADHD (Bishop et al., 2017; Cortese et al., 2026; Hull et al., 2020; Lai et al., 2015; Norbury et al., 2016). These observations suggest that developmental trajectories and response to intervention are not equally integrated into diagnostic reasoning across disorders, settings, and clinicians.

Time thus becomes a central dimension of assessment. Observing changes under intervention reveals trajectories not captured by single time-point reports: some converge toward expected developmental profiles, suggesting transient immaturity, whereas others remain persistently atypical despite appropriate adjustments, indicating more enduring constraints (Nigg, 2017). Within this framework, early intervention serves not only a therapeutic role but also a diagnostic function, enabling targeted environmental adjustments and helping distinguish transient difficulties from more persistent constraints.

Diagnosis in NDDs therefore differs from many somatic conditions, as clinicians are often required to infer the persistence of a developmental disorder from observations made within an evolving and inherently heterogeneous system.

## 3 Developmental and neurobiological foundations

Research in neuroscience shows that brain maturation is neither linear nor synchronous across regions (Gogtay et al., 2004; Lenroot and Giedd, 2006; Vijayakumar et al., 2016). These findings move beyond stage-based models and point to a continuous and dynamic organization of development shaped by interactions with the environment. Brain maturation follows a prolonged and hierarchical trajectory, broadly characterized by a caudo-rostral progression. Sensory and motor regions reach functional maturity early, whereas associative and prefrontal regions continue to develop throughout childhood, adolescence, and into early adulthood (Casey et al., 2016). This temporal organization shows substantial intra- and inter-individual variability (Stiles and Jernigan, 2010).

From a clinical perspective, this variability implies that the apparent absence of specific skills at a given time may reflect a transient stage of an ongoing maturation process rather than the stable expression of a disorder. Within this framework, high levels of plasticity during childhood allow adaptation to learning experiences and environmental conditions, but also imply increased sensitivity to stimulation and contextual influences. At the same time, this plasticity unfolds within constrained trajectories, shaped by sensitive periods and increased vulnerability to disruption (Kolb and Gibb, 2014).

In parallel, environmental demands evolve progressively. Educational, social, and behavioral contexts become increasingly complex, requiring higher levels of regulation, adaptation, and autonomy. The visibility of difficulties therefore depends not only on an underlying vulnerability, but also on the mismatch between individual resources and environmental demands (Casey et al., 2016). Some difficulties may remain barely detectable in low-demand or highly structured contexts and only emerge when this balance is no longer sufficient. Clinical manifestations such as impulsivity, rigidity, or disorganization must therefore be interpreted relative to developmental expectations, allowing a distinction between normative variability and significant deviation.

These dynamics illustrate the structuring role of time in neurodevelopment. They are consistent with work in developmental neurology emphasizing the ontogenetic adaptation of the developing nervous system and the importance of age-appropriate assessment, as developmental changes influence the interpretation of neurological and behavioral findings (Prechtl, 2001). Similarly, Marschik et al. (2017) proposed a framework based on age-specific constellations of developmental features that may facilitate earlier detection and characterization of neurodevelopmental disorders. These concepts may also help explain relative age effects in educational systems, where children who are youngest within a school cohort appear more likely to receive a diagnosis of attention deficit hyperactivity disorder, possibly reflecting relative developmental immaturity rather than qualitative differences in functioning (Caye et al., 2020). These findings highlight the risk of misinterpreting typical developmental variation as pathological when the temporal dimension is insufficiently considered.

These observations suggest that time is not merely a background dimension of neurodevelopment but may itself constitute a clinically informative variable whose interpretation necessarily depends on developmental timing and age-appropriate expectations. If developmental heterogeneity is an intrinsic characteristic of neurodevelopment, an important question is how this temporal dimension can be incorporated into diagnostic reasoning and clinical decision-making.

## 4 Time and developmental trajectories in diagnostic reasoning

**FIGURE 1:**
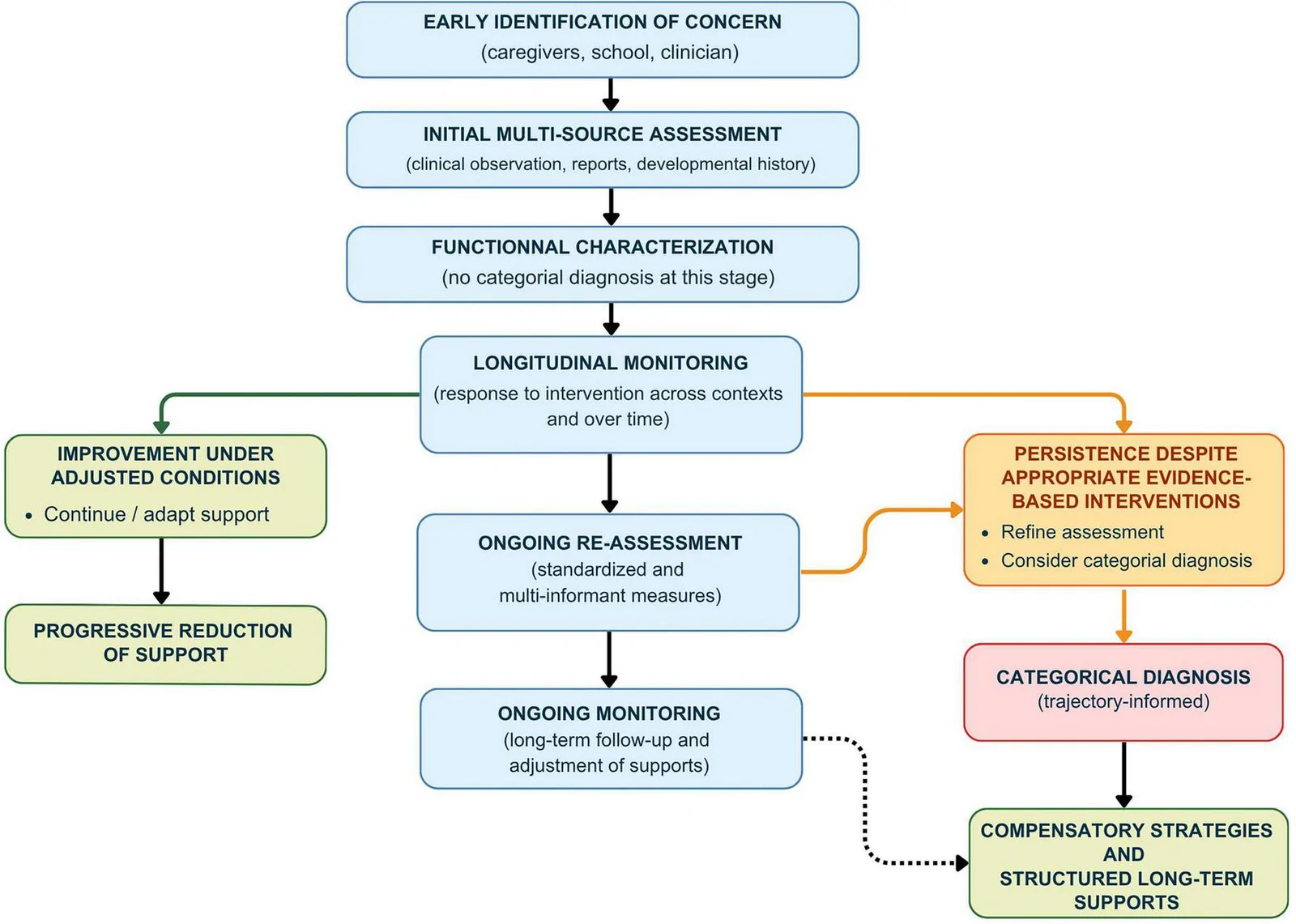
Illustration of a trajectory-informed clinical process for early identification, intervention, and diagnostic reasoning in neurodevelopmental disorders. Following early identification, functional characterization guides intervention without requiring a prior diagnosis. Longitudinal monitoring and repeated assessment evaluate response to intervention across contexts; improvement supports adaptation and reduction of support, whereas persistence despite appropriate evidence-based interventions may prompt re-assessment and contribute to trajectory-informed diagnostic reasoning and long-term support planning.

Building on this framework, and consistent with approaches seeking to characterize age-specific developmental constellations (Marschik et al., 2017), longitudinal follow-up provides a framework for analyzing developmental trajectories. It allows clinicians to examine both spontaneous changes in functioning and their sensitivity to environmental adjustments (Casey et al., 2016; Johnson, 2011; Karmiloff-Smith, 1998), and may be implemented as a clinical process, as illustrated in Figure 1. In practice, it complements assessments based on behavioral and functional manifestations, whose interpretation remains sensitive to context and informant discrepancies (Achenbach et al., 1987; De Los Reyes et al., 2015).

Current diagnostic reasoning relies primarily on current observations and retrospectively reconstructed accounts obtained in naturalistic settings rather than on prospective observations of developmental trajectories under structured environmental adjustments. Understanding how difficulties emerge, evolve, or persist as environmental conditions change therefore remains essential for diagnostic reasoning (Frith, 2012; Johnson, 2011). Within this perspective, interventions and environmental adjustments are not only therapeutic; they also provide opportunities to observe developmental responses, revealing the malleability or persistence of underlying vulnerabilities (Bishop et al., 2017; Lord et al., 2020).

Developmental language trajectories provide a useful illustration of this issue. Approximately 10%–20% of 2-year-old children exhibit late language emergence, yet longitudinal studies indicate that nearly 75%–85% subsequently attain language abilities within the average range, whereas only a minority present persistent difficulties at school age consistent with developmental language disorder (Norbury et al., 2016; Nouraey et al., 2021). Such findings illustrate how the clinical significance of early developmental difficulties often depends less on their initial presence than on their developmental trajectory over time (Bishop et al., 2017; McGregor, 2020). When language difficulties persist, however, their developmental consequences may evolve in ways that complicate diagnostic interpretation. A child described as “daydreaming” and failing to complete tasks may initially suggest attentional difficulties, while language comprehension deficits leading to cognitive overload and disengagement may better account for the observed behaviors. Likewise, persistent language difficulties may also complicate early clinical evaluation of autism spectrum disorder, where language delay, compensatory strategies and evolving social communication profiles may contribute to diagnostic uncertainty and under-identification in some individuals (Hull et al., 2020; Lai et al., 2015). Across these developmental trajectories, longitudinal follow-up and targeted interventions help distinguish transient developmental variations from more persistent neurodevelopmental constraints. Rather than relying solely on their initial presentation, clinicians may therefore better interpret early developmental difficulties by examining how they evolve over time and respond to environmental adaptations (Lord et al., 2020).

This perspective aligns with principles already embedded within existing diagnostic frameworks and clinical practices. For example, DSM-5-TR criteria for specific learning disorder require the persistence of difficulties despite targeted interventions (American Psychiatric Association, 2022). Developmental expectations and adaptive functioning also play a central role in the assessment of intellectual developmental disorder, while repeated observations may help refine early clinical evaluations of autism spectrum disorder. Nevertheless, clinical practices remain heterogeneous regarding how developmental trajectories and response to intervention are incorporated into diagnostic reasoning, with considerable variability persisting between professionals, settings, and healthcare systems. The present perspective therefore aims less at proposing a disorder-specific model than at encouraging a more explicit, developmentally informed and harmonized consideration of supported trajectories within neurodevelopmental diagnostic reasoning.

Within this framework, the key issue is not only whether difficulties are present, but how stable, malleable, and context-dependent they are. Several developmental indicators may contribute to such longitudinal characterization. Among them, executive functions appear particularly informative because they provide insight into how regulatory capacities evolve in response to increasing demands and may reveal compensatory strategies that mask underlying vulnerabilities (Hull et al., 2017; Livingston and Happé, 2017). Interpreting these dynamics requires integrating observations across contexts and informants within a coordinated perspective (Achenbach et al., 1987; De Los Reyes et al., 2015).

Thus, longitudinal follow-up does not merely document change. It enables clinicians to evaluate the stability, malleability, and conditions of expression of developmental difficulties, providing a structured basis for distinguishing transient variations from more persistent neurodevelopmental disorders (Johnson, 2011).

## 5 Executive functions as illustrative developmental markers

Several developmental domains, including language, motor coordination, and adaptive functioning, may inform the longitudinal characterization of developmental trajectories. Executive functions were selected here as a particularly informative developmental marker because an increasing body of literature conceptualizes them as a transdiagnostic developmental construct involved across neurodevelopmental, psychiatric, neurological, and genetic conditions rather than as disorder-specific deficits (Nigg, 2017; Roy and Fournet, 2021; Sadozai et al., 2024; Zelazo, 2020). Executive dysfunction has been associated with heterogeneous manifestations affecting social, emotional, and behavioral functioning (Nigg, 2017). Executive alterations have been documented across a broad range of neurodevelopmental conditions, including autism spectrum disorder and developmental language disorder, as well as in children born preterm, following acquired brain injury, after prenatal exposure to adverse environmental factors, and in genetic conditions such as neurofibromatosis type 1, supporting their characterization as transdiagnostic vulnerabilities affecting regulatory systems (Aarnoudse-Moens et al., 2009; Anderson et al., 2011; Remaud et al., 2025). Their involvement in compensatory strategies, adaptive functioning and later developmental outcomes further supports their relevance for examining developmental trajectories over time (Hull et al., 2017; Moffitt et al., 2011; Morgan et al., 2019).

Executive functions emerge during early childhood along a prolonged trajectory (Best and Miller, 2010; Diamond, 2013). Early forms are observable from the preschool years and become progressively more specialized with the maturation of fronto-subcortical networks (Casey et al., 2005; Diamond, 2013; Zelazo, 2020). Difficulties often become more visible when environmental demands exceed regulatory capacities, with some children functioning adequately in structured environments but struggling when greater autonomy, flexibility, or emotional regulation is required.

Rather than constituting disorder-specific markers, they can be viewed as a developmental window onto regulatory processes that unfold across contexts and over time (Nigg, 2017; Roy and Schoentgen, in press). Combined with their prolonged maturation, executive functions provide insight into the dynamic balance between brain maturation, individual resources, compensatory strategies, and environmental demands. Their expression may vary across contexts and developmental stages, making point-in-time interpretations uncertain. Repeated assessments may therefore help identify mechanisms of compensation, masking, or behavioral disorganization and support a more integrated interpretation of functioning over time.

**FIGURE 2:**
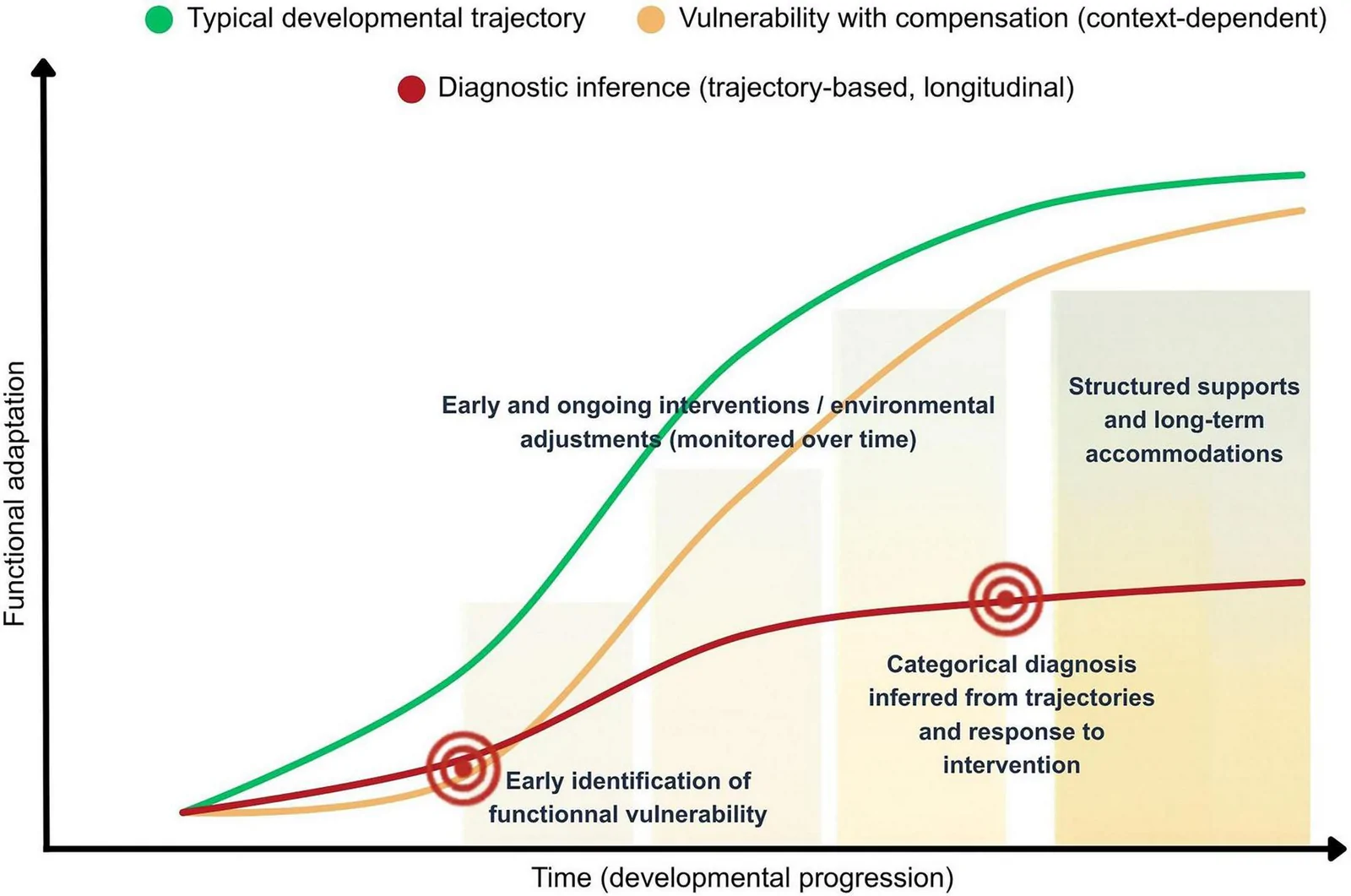
Developmental trajectories and trajectory-informed diagnostic inference in neurodevelopmental disorders. Schematic representation of developmental trajectories of functional adaptation: typical (green), compensatory (orange), and persistent vulnerability (red). Early identification enables intervention prior to categorical diagnosis, while diagnostic inference may emerge from the longitudinal analysis of trajectories and their response to environmental adjustments.

Within this developmental framework, improvement following structured support may suggest a contextual imbalance, whereas persistence despite appropriate interventions may support the hypothesis of an underlying neurodevelopmental constraint. Tracking executive functioning over time and across environmental contexts may help identify persistent deviations suggestive of more durable neurodevelopmental constraints, as illustrated in Figure 2.

Thus, executive functions may represent one developmental lens through which the stability, malleability, and contextual expression of difficulties can be interpreted over time, complementing broader longitudinal approaches to diagnostic reasoning.

## 6 Clinical implications for longitudinal diagnostic reasoning

The framework developed in this article has implications for both clinical practice and, more broadly, for research. It calls for a re-examination of the relationship between early identification and diagnosis, which remain closely linked in current practice. While early detection has become a priority, diagnostic processes do not always fully account for the dynamic, non-linear, and context-dependent nature of development, contributing to tensions between timely intervention and robust diagnostic interpretation.

Cross-sectional assessments remain essential for characterizing functioning at a given time. However, no single cross-sectional observation, whether based on clinical consultation, standardized assessment, or behavioral report, can fully capture the developmental processes underlying neurodevelopmental disorders. Their diagnostic value therefore remains limited if not interpreted in relation to temporal evolution and responsiveness to environmental adjustments. This highlights the need to complement clinical evaluations with structured longitudinal monitoring supported by repeated standardized assessments. This perspective does not imply delaying diagnosis but reframing it as an iterative and developmentally informed process. Early intervention can be initiated based on functional vulnerabilities, while longitudinal observation refines diagnostic interpretation.

Clinical judgment remains central, but its robustness depends on integrating longitudinal observations collected across multiple time points, contexts and standardized assessment methods. Repeated standardized assessments provide a stable reference across time and between clinicians. Such monitoring may combine ecological observations, multi-informant questionnaires, adaptive functioning measures, and repeated assessments selected according to developmental stage, clinical hypotheses, and intervention targets. Within this perspective, comprehensive neuropsychological evaluation may constitute one useful component of a longitudinal assessment framework by integrating standardized performance measures, ecological observations, informant reports, and repeated assessments over time (Coghill et al., 2014). In this respect, developmental assessment may be understood in a manner analogous to growth monitoring: a single measurement below normative expectations does not in itself warrant concern, whereas repeated observations showing persistent deviation across time provide a more reliable basis for clinical interpretation and decision-making.

From a clinical perspective, this approach supports earlier intervention without requiring immediate categorical certainty. By integrating the evolution and context-dependence of clinical manifestations into decision-making, it may reduce both delays in support and premature diagnostic labeling. It is also consistent with emerging models of care that emphasize needs-led support while preserving the imperative for early intervention (NHS England, 2025).

## 7 Limitations and future directions

The present perspective does not propose a new diagnostic framework or seek to redefine existing classifications. Rather, it aims to make more explicit practices that already incorporate developmental trajectories, intervention-supported observations, and longitudinal reasoning in the assessment of neurodevelopmental disorders. Several challenges nevertheless deserve consideration.

A first challenge concerns the interpretation of time itself. This perspective does not advocate delaying diagnosis or access to intervention while awaiting greater certainty. On the contrary, it fully supports early identification efforts through improved awareness and training of healthcare professionals, early childhood practitioners, educators, and families. Within this perspective, intervention may, in some situations, be partially decoupled from categorical diagnosis, allowing interventions to be initiated based on functional vulnerabilities and developmental needs while longitudinal follow-up informs diagnostic interpretation. Time should therefore be considered as an active clinical process involving targeted interventions, environmental adaptations, and repeated assessments rather than as a passive waiting period. The timing and modalities of follow-up should remain informed by developmental stage, clinical hypotheses, intervention targets, and professional judgment.

A second challenge concerns applicability. Access to multidisciplinary assessments, trained professionals, and specialized services remains uneven and may limit the feasibility of structured longitudinal monitoring. Standardized diagnostic classifications do not in themselves guarantee homogeneous clinical practices. Even within healthcare systems operating under common DSM or ICD frameworks, substantial variability persists regarding expertise, thresholds for clinically significant impairment (Hull et al., 2020; Norbury et al., 2016). Greater harmonization of these practices may therefore represent an important objective if diagnostic reasoning is to better reflect current knowledge regarding brain maturation, developmental variability, and responsiveness to intervention (Regier et al., 2013).

This perspective also has implications for research practices. Neurodevelopmental research cohorts are often assembled through multiple clinical settings involving differences in diagnostic procedures, assessment strategies, and intervention histories. Such variability may contribute to heterogeneous cohort composition, increasing phenotypic heterogeneity and complicating the identification of robust and reproducible neurobiological findings (Mottron and Bzdok, 2020; O’Connor et al., 2026). These concerns have been raised in autism research and discussed in developmental language disorder and ADHD, where variability in diagnostic thresholds, age at diagnosis, clinical pathways and the identification of less prototypical presentations may all influence cohort composition (Bishop et al., 2017; Norbury et al., 2016; Setyawan et al., 2018). More generally, compensatory mechanisms and less overt presentations across neurodevelopmental disorders may further support the integration of developmental trajectories into diagnostic reasoning, but also into the design and interpretation of neurodevelopmental research (Livingston and Happé, 2017).

Finally, no consensus currently exists regarding how supported developmental trajectories should be incorporated into diagnostic reasoning. Future research should aim to identify reliable developmental indicators that may help clinicians interpret individual trajectories, including their stability, malleability, and responsiveness to intervention. Such efforts may strengthen the clinician’s role in integrating developmental knowledge, contextual information, and longitudinal observations into individualized diagnostic formulations rather than relying solely on symptoms observed at a single time point.

## 8 Conclusion

Neurodevelopmental disorders are inherently developmental conditions. Integrating developmental trajectories, contextual factors, and responsiveness to intervention into clinical reasoning may help reconcile the need for early support with the requirements for robust diagnostic interpretation.

In neurodevelopment, diagnosis is not defined by symptoms alone, but by how they evolve under changing environmental conditions. Time is not merely a context for observation, but a core component of diagnostic reasoning. It is the interpretation of a trajectory, not the immediate reading of a state, that may provide the most meaningful understanding of neurodevelopmental difficulties.

## Data Availability

The original contributions presented in this study are included in this article/supplementary material, further inquiries can be directed to the corresponding author.
